# United States experience with primary HIFU therapy for patients with low-risk prostate cancer: results of the Enlight trial

**DOI:** 10.1186/2050-5736-3-S1-P85

**Published:** 2015-06-30

**Authors:** Cary Robertson, Anthony Sliwinski, Eric Wallen, William Orovan, Inderbir Gill, John Ward

**Affiliations:** 1 Duke University Medical Center, Durham, North Carolina, United States; 2 Virginia Urology, Richmond, Virginia, United States; 3 University of North Carolina, Chapel Hill, North Carolina, United States; 4 McMaster University, Hamilton, Canada; 5 University of Southern California, Los Angeles, California, United States; 6 MD Anderson Cancer Center, Houston, Texas, United States

## Background/introduction

High Intensity Focused Ultrasound (HIFU) is a non-invasive treatment for localized prostate cancer. The purpose of this study was to investigate the safety and effectiveness of HIFU as a monotherapy for the initial treatment of low-risk prostate cancer (PCa) in the United States.

## Methods

An Investigational Device Exemption trial received local IRB approval at thirteen sites in the United States and Canada. Subjects with untreated low-risk localized PCa were recruited and treated with single session monotherapy HIFU without adjuvant TURP or hormone ablation. Repeat HIFU procedures were not permitted. Subjects were followed at 1 and 3 months and every three months thereafter. The primary endpoint was 24 month biochemical freedom from failure (the “Phoenix” definition). Subjects underwent biopsy for cause (rising PSA) or at the end of study (24 months). Adverse events were assessed at each postoperative visit and reported as mild, moderate or severe and related to the device or procedure at 24 months.

## Results and conclusions

A total of 135 subjects were prospectively enrolled. Mean age and PSA at treatment (± SD) was 64.1 ± 6.7 years and 4.6 ± 2.4 ng/ml, respectively. The Gleason grade and stage were 6 and T1c for 97% and 81% of subjects, respectively. A PSA nadir < 0.5 ng/ml was achieved in 74.1% of subjects. The primary biochemical endpoint was achieved by 90.5% (95% CI: 85.2% - 95.8%) of subjects. Freedom from positive biopsy was 97/135 (72%) at two years. The erectile dysfunction rate was 38%, urinary incontinence: 3%, urinary retention: 3% and stricture: 1%. No fistulae were observed. Both the local (biopsy) control and the biochemical survival rates are promising following HIFU which was utilized as a single session monotherapy without any adjuvants. The adverse event profile demonstrates promising erectile function preservation and low rates of long term morbidity. These results complement published long term outcomes from Europe, where HIFU is utilized in combination therapy, in repeat treatments, and as salvage treatment. Results from this study show that HIFU appears to be a safe and efficacious primary therapy for localized prostate cancer.

